# COVID-19 in pediatric cancer patients is associated with treatment interruptions but not with short-term mortality: a Polish national study

**DOI:** 10.1186/s13045-021-01181-4

**Published:** 2021-10-11

**Authors:** Jadwiga Węcławek-Tompol, Zuzanna Zakrzewska, Olga Gryniewicz-Kwiatkowska, Filip Pierlejewski, Ewa Bień, Agnieszka Zaucha-Prażmo, Olga Zając-Spychała, Anna Szmydki-Baran, Agnieszka Mizia-Malarz, Wioletta Bal, Małgorzata Sawicka-Żukowska, Agnieszka Kruk, Tomasz Ociepa, Anna Raciborska, Agnieszka Książek, Tomasz Szczepański, Jarosław Peregud-Pogorzelski, Maryna Krawczuk-Rybak, Radosław Chaber, Michał Matysiak, Jacek Wachowiak, Ninela Irga-Jaworska, Wojciech Młynarski, Bożenna Dembowska-Bagińska, Walentyna Balwierz, Agnieszka Matkowska-Kocjan, Bernarda Kazanowska, Jan Styczyński, Marek Ussowicz

**Affiliations:** 1 Department and Clinic of Pediatric Oncology, Haematology and Bone Marrow Transplantation, Wroclaw Medical University, Borowska 213, 50-556 Wroclaw, Poland; 2 Department of Pediatric Oncology and Hematology, Institute of Pediatrics, Jagiellonian University, Collegium Medicum, Kraków, Poland; 3 Department of Pediatric Oncology, Children’s Memorial Health Institute, Warsaw, Poland; 4 Department of Pediatrics, Hematology and Oncology, Medical University of Lodz, Lodz, Poland; 5 Department of Pediatrics, Hematology and Oncology, Medical University of Gdansk, Gdansk, Poland; 6 Department of Pediatric Hematology, Oncology and Transplantology, Medical University of Lublin, Lublin, Poland; 7 Department of Pediatric Oncology, Hematology and Transplantology, University of Medical Sciences, Poznan, Poland; 8 Department of Oncology, Pediatric Hematology, Transplantology and Pediatrics, Children’s Hospital, Medical University of Warsaw, Warsaw, Poland; 9 Department of Pediatrics, Medical University of Silesia, Katowice, Poland; 10 Clinic of Pediatric Oncology and Hematology, State Hospital 2 in Rzeszow, Rzeszow, Poland; 11 Department of Paediatrics, Institute of Medical Sciences, University of Rzeszow, Rzeszow, Poland; 12 Department of Pediatric Oncology and Hematology, Medical University of Bialystok, Białystok, Poland; 13 Department of Paediatrics, Paediatric Oncology and Immunology, Pomeranian Medical University, Szczecin, Poland; 14 Department of Oncology and Surgical Oncology for Children and Youth, Institute of Mother and Child, Warsaw, Poland; 15 Department of Pediatric Hematology and Oncology, Zabrze, Medical University of Silesia, Katowice, Poland; 16 Department and Clinic of Pediatric Infectious Diseases, Wroclaw Medical University, Wroclaw, Poland; 17 Department of Pediatric Hematology and Oncology, Jurasz University Hospital, Collegium Medicum Nicolaus Copernicus University Torun, Bydgoszcz, Poland; 18 Department of Pediatrics, Hemato-Oncology and Gastroenterology, Pomeranian Medical University, Szczecin, Poland

**Keywords:** Pediatric, SARS-CoV-2, COVID-19, Chemotherapy

## Abstract

**Background:**

Coronavirus disease 2019 (COVID-19) caused by severe acute respiratory syndrome coronavirus 2 (SARS-CoV-2) currently constitutes the leading and overwhelming health issue worldwide. In comparison with adults, children present milder symptoms, with most having an asymptomatic course. We hypothesized that COVID-19 infection has a negative impact on the continuation of chemotherapy and increases nonrelapse mortality.

**Material and methods:**

This study was performed to assess the course of SARS-CoV-2 among children with hematological or oncological malignancies and its impact on cancer therapy. Records of SARS-CoV-2 infection in 155 children with malignancies from 14 Polish centers for pediatric hematology and oncology were collected and analyzed.

**Results:**

SARS-CoV-2 replication was observed in 155 patients. Forty-nine patients were symptomatic, with the following being the most common manifestations: fever (31 patients), gastrointestinal symptoms (10), coryza (13), cough (13) and headache (8). In children who were retested, the median time of a positive PCR result was 16 days (range 1–70 days), but 12.7% of patients were positive beyond day + 20. The length of viral PCR positivity correlated with the absolute neutrophil count at diagnosis. Seventy-six patients did not undergo further SARS-CoV-2 testing and were considered convalescents after completion of isolation. Antibiotic therapy was administered in 15 children, remdesivir in 6, convalescent plasma in 4, oxygen therapy in 3 (1—mechanical ventilation), steroids in 2, intravenous immunoglobulins in 2, and heparin in 4. Eighty patients were treated with chemotherapy within 30 days after SARS-CoV-2 infection diagnosis or were diagnosed with SARS-CoV-2 infection during 30 days of chemotherapy administration. Respiratory symptoms associated with COVID-19 and associated with oxygen therapy were present in 4 patients in the study population, and four deaths were recorded (2 due to COVID-19 and 2 due to progressive malignancy). The probability of 100-day overall survival was 97.3% (95% CI 92.9–99%). Delay in the next chemotherapy cycle occurred in 91 of 156 cases, with a median of 14 days (range 2–105 days).

**Conclusions:**

For the majority of pediatric cancer patients, SARS-CoV-2 infection does not result in a severe, life-threatening course. Our data show that interruptions in therapy are common and can result in suboptimal therapy.

**Supplementary Information:**

The online version contains supplementary material available at 10.1186/s13045-021-01181-4.

## Introduction

Coronavirus disease 2019 (COVID-19) caused by the new β-coronavirus severe acute respiratory syndrome coronavirus 2 (SARS-CoV-2) currently constitutes the leading and overwhelming health issue worldwide. By the date of submission of this article, 173,217,070 COVID-19 cases and 3,725,016 related deaths had been confirmed worldwide [1]. SARS-CoV-2 can replicate in different tissues, with the respiratory system and gastrointestinal tract being the most important systems involved. SARS-CoV-2 infection has two different phases. In the first phase, viral replication occurs, and in severe cases, it is followed by a second phase characterized by a dysregulated immune response with tissue damage [2, 3]. Mechanisms underlying lung injury in both phases are different but can result in irreversible lung damage and death due to respiratory failure. COVID-19 severity clearly depends on the age of the individual—children present milder symptoms in comparison with the adult population. Mortality among children with COVID-19 is currently estimated at less than 0.03% [4]. Furthermore, the death rate is low in the youngest age group, but age below 1 month, male sex, pre-existing medical conditions, and presence of lower respiratory tract infection signs or symptoms at presentation are associated with higher complication rates [5–7]. For immunocompromised children, as many as 77% show an asymptomatic course of SARS-CoV-2 infection [8].

Polish pediatric oncology and hematology centers provide care for 1850 newly diagnosed patients each year, including 1300 children with cancer. This study was performed to assess the course of SARS-CoV-2 infection among Polish children with hematological or oncological malignancies who have been actively treated with systemic therapy and its impact on the timing of cancer therapy. We hypothesized that COVID-19 infection increases nonrelapse mortality and has a negative impact on the continuation of chemotherapy.

### Statistical analysis

The endpoint was overall survival (OS), which was defined as the time from SARS-CoV-2 diagnosis to death or the last report for patients with no events, and cumulative incidence (CI) of PCR test negativity. OS and CI curves were estimated using the Kaplan–Meier method. Correlation analysis between the duration of viral replication and blood counts was carried out with Spearman’s test. Cox modeling was adopted to estimate hazard ratios for OS and CI, considering *P* < 0.2 as significant.

The statistical analysis and data formatting for presentation were performed with GraphPad Prism software (GraphPad Software, La Jolla, CA, USA) and STATISTICA 13.3 (TIBCO Software Inc. 2017, STATISTICA, version 13, Dell, OK, USA).

## Patients and methods

Data from 14 Polish centers for pediatric hematology and oncology were collected. In the study period (March 2020–February 2021), approximately 1200 new pediatric cancer diagnoses were made and approximately 2500 children were treated with chemotherapy. All patients hospitalized at Polish pediatric hematology and oncology centers were routinely tested for SARS-CoV-2 at admission (since June 2020) and in cases of suspected COVID-19 (any time, at physician discretion). In the majority of centers, testing of parents hospitalized with their children was carried out. Diagnostics of SARS-CoV-2 replication were performed using different polymerase chain reaction (PCR) tests identifying the presence of viral particles at the level of nucleic acid testing (NAT). Since 2021, “quick” antigen testing has been additionally offered as a screening test; in symptomatic negative cases, verification with NAT testing is recommended. Nasopharyngeal swabs were used as the material for viral testing. Patients who tested positive were isolated at home or were referred to specialized infectious disease units if symptomatic or requiring medical procedures. The time of isolation of patients with SARS-CoV-2 infection depended on the current law regulations in Poland, which changed during the course of the pandemic. Until 1 September 2020, there had been a requirement of 2 consecutive negative PCR results after at least 14 days of isolation; since 2 September 2020, Poland has adopted European Centre for Disease Prevention and Control (ECDC) guidelines on the ending of COVID-19 isolation: resolution of fever for at least three days and clinical improvement of symptoms other than fever and 10 days after the onset of symptoms in immunocompetent patients or 20 days after the onset of symptoms in immunocompromised patients [9, 10]. The data collection ended on 1 March 2021. The patient or parent/legal guardian provided written informed consent for the treatment and the analysis of clinical data. Ethical approval was waived by the local Ethics Committee of Wroclaw Medical University in view of the retrospective nature of the study because all procedures were performed as a part of routine care.

## Results

**Fig. 1:**
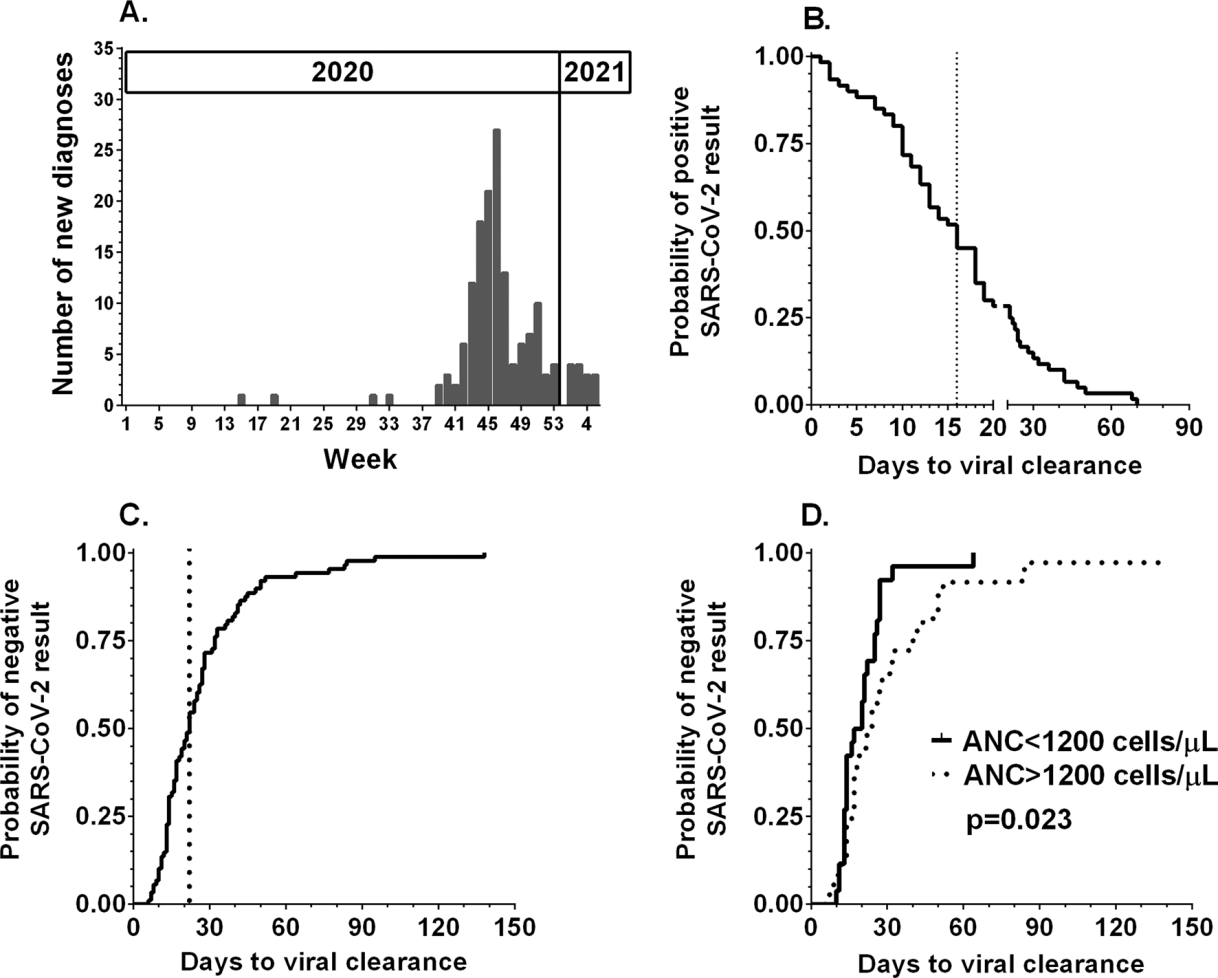
**A** Incidence of SARS-CoV-2 infection in pediatric cancer patients in 2020 and early 2021. **B** The duration of SARS-CoV-2 replication was calculated as the interval between the first and last positive results. The dotted line marks day 16, which was the median length of PCR positivity. **C** Time to clearance of SARS-CoV-2 replication was calculated as the interval between the first positive and first negative results. The dotted line marks the median time to a first negative result on day 22. **D** Comparison of the probability of SARS-CoV-2 negativity between patients with absolute neutrophil counts (ANCs) below and above 1200/µL at diagnosis

For this study, records of SARS-CoV-2 episodes in 155 patients were collected. The patient characteristics are shown in Table 1. The continuous wave of SARS-CoV-2 diagnoses was noted since the 39th week of 2020, and the maximum weekly number of new diagnoses was 27, which declined since the 50th week of 2020 (Fig. 1A).

**Table 1:** Patient characteristics

Category	
Age in months median (range)	71 (1–207)
Sex (male [M]: female [F])	93 M:62 F

Diagnosis	Patients number	Delay in chemotherapy: median (range)
Acute lymphoblastic leukemia (ALL)	52	13 (0–105)
CNS tumor	30	14 (0–60)
Soft tissue sarcoma (STS)	16	12 (0–26)
Neuroblastoma	15	15 (0–44)
Hodgkin lymphoma (HL)	10	12 (0–15)
Renal tumor	8	17 (12–19)
Acute myeloid leukemia (AML)	7	17 (0–56)
Non-Hodgkin lymphoma (NHL)	4	17 (0–20)
Osteosarcoma	4	7 (0–14)
Retinoblastoma	2	7 (n/a)
Other (EWS, GCT, HCC, LCH, NET, SPN)	7	14 (10–27)

CNS, central nervous system; EWS, Ewing’s sarcoma; GCT, germ cell tumor; HCC, hepatocellular carcinoma; LCH, Langerhans histiocytosis; NET, neuroendocrine tumor; SPN, solid pseudopapillary neoplasm

### Testing policy and replication length

Diagnosis was established with PCR-NAT in 138 patients, both PCR-NAT and an antigenic test in 12, and with antigenic test in only 5. In 79 cases, the patient was tested again after SARS-CoV-2 diagnosis for PCR test negativity. The testing was performed at different intervals; on average, the median was 14 days but ranged from 2 to 133 days. A single SARS-CoV-2 result was obtained for 36 patients, who were tested again after a median of 15 days (range 6 to 82 days). Among patients who had subsequent positive PCR results, the median time of viral replication was 16 days (range 1–70 days); it should be noted that 25% of patients had positive results beyond day + 20 (Fig. 1B). The length of PCR positivity correlated with the absolute neutrophil count at diagnosis (Spearman rank *r* = 0.2642, 95% CI 0.04207 to 0.4614, *p* = 0.0172) but not with age at diagnosis, sex, whole blood count (WBC), lymphocyte or monocyte counts. The median time until laboratory-confirmed viral clearance was 22 days (range 6–138 days) (Fig. 1C). The time of PCR negativity correlated with the absolute neutrophil count at diagnosis (Spearman rank *r* = 0.2547, 95% CI − 0.002269 to 0.4801, *p* = 0.0457) but not with age at diagnosis, sex, WBC, lymphocyte or monocyte counts. For patients with absolute neutrophil counts below or above 1200 per µL, we calculated a median of 18 versus 23 days until a negative PCR result (*p* = 0.023, Fig. 1D). The remaining 76 patients were not further tested for SARS-CoV-2 and were considered to be in the convalescent stage after completion of the recommended time of isolation.

### Symptoms

**Fig. 2:**
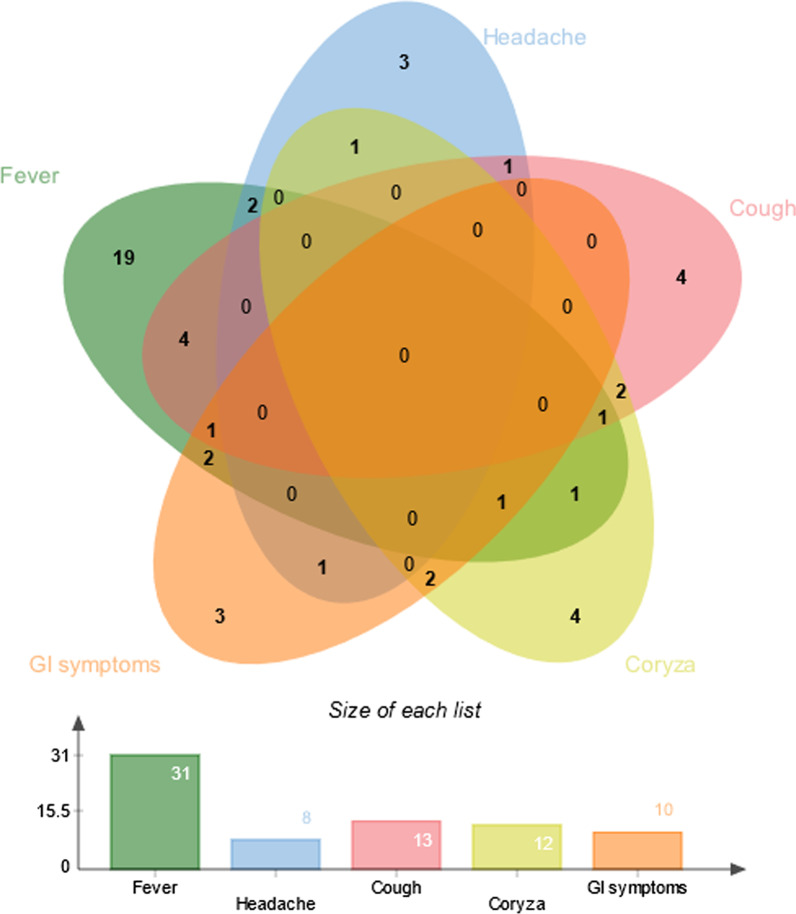
Venn diagram presenting the symptomatology of SARS-CoV-2 infection. The five most common symptoms were observed in different combinations in 49 patients

**Fig. 3:**
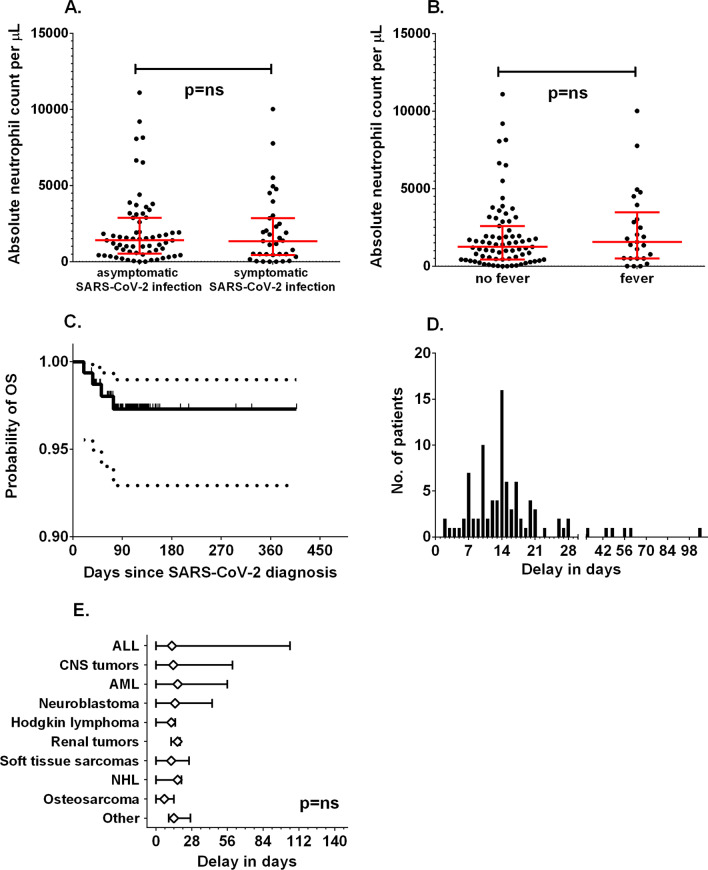
Comparison of absolute neutrophil counts between patients with symptomatic or asymptomatic manifestations (**A**) and in those with fever at diagnosis (**B**). **C** The overall survival of children with SARS-CoV-2 infection. Dotted lines represent the 95% CI. **D** The distribution of delay times in chemotherapy among pediatric patients. **E** Delay times among different diagnosis groups; diamonds represent median delay and whiskers minimum to maximum range

Symptoms were noted in 49 patients. The most common manifestation was fever, in 31 patients, followed by gastrointestinal symptoms in 10, coryza in 12, cough in 13 and headache in 8; symptoms were present in different combinations (Fig. 2). At diagnosis, complete blood counts were available for 100 patients. Overall, WBC and absolute lymphocyte, monocyte and neutrophil counts at SARS-CoV-2 diagnosis were not associated with the presence of any symptoms or, in particular, fever incidence (Fig. 3A, B).

### Treatment and survival

In the group analyzed, antibiotic therapy was administered in 15 children, remdesivir in 6, convalescent plasma in 4, oxygen therapy in 3 (1—mechanical ventilation), steroids in 2, intravenous immunoglobulins in 2, and heparin in 4. The probability of 100-day overall survival was 97.3% (95% CI 92.9–99%) (Fig. 3C). In the whole study group, four deaths were recorded (2 due to COVID-19 and 2 due to progressive malignancy).

### Chemotherapy

Eighty patients were treated with chemotherapy within 30 days after SARS-CoV-2 infection diagnosis or were diagnosed with SARS-CoV-2 infection within 30 days of chemotherapy administration. Chemotherapy was administered within 30 days of SARS-CoV-2 diagnosis in 12 patients, but chemotherapy was interrupted in 5. In this subgroup, 2 patients with acute lymphoblastic leukemia (ALL; UPN 3 and UPN 25) developed COVID-19 pneumonia and required oxygen therapy but survived without pediatric intensive care unit (PICU) transfer; their cancer treatment continued after recovery. In addition, UPN 25 was treated for acute gastrointestinal bleeding.

In 68 patients, chemotherapy was started within 30 days after SARS-CoV-2 infection was diagnosed. In this subgroup, 1 patient (UPN 147) was hospitalized for 32 days in the PICU, and 3 deaths were recorded: UPN 147 with ALL treated with protocol I, and UPN 148 with early AML relapse after allogeneic hematopoietic cell transplantation (HCT) and treated with Ida-FLA and venetoclax died due to respiratory failure, and UPN 145 with central nervous system tumor (CNS) died of disease progression. Table 2 presents the chemotherapy cycles in both groups in the analyzed period.

**Table 2:** Systemic therapy during the period of SARS-CoV-2 infection

Diagnoses	Patients with SARS-CoV-2 diagnosis within 30 days prior to chemotherapy		Patients with SARS-CoV-2 diagnosis within 30 days after the chemotherapy	
	Number of patients	Chemotherapy protocol—number of patients	Number of patients	Chemotherapy protocol—number of patients
Acute lymphoblastic leukemia	7	Protocol I—1; protocol II—6	20	Protocol I—3; protocol M—3; protocol HR—3; protocol II—7; maintenance therapy—3; relapsed—1
CNS tumor	1	VCR + VP-16 + CTX—1	15	VCR + cisplatin—3; VCR—2; VCR + VP-16 + CTX—1; VCR + cisplatin + CCNU—1; protocol 4 (week 8; week 29)- 2; protocol 3 (cycle 2)—1; VBL—2; Protocol 1 HRG—1; temozolomide + cisplatin—1; radiotherapy—1
Soft tissue sarcoma	–	–	13	IVAd3-1; VCR-ADM-CTX-1; gemcitabine + vinorelbine—1; I2VAdr-1; ACCTTIVE-1; I3VActd-1; idarubicin + trofosfamide -1; radiotherapy—2; I2VE-1; I2VA-1; TECC -1; VAC—1
Neuroblastoma	2	Topotecan + dacarbazine—1; topotecan—1	2	COJEC-A—1; radiotherapy—1
Hodgkin lymphoma	–	–	5	AVD—2; COPDAC—2; DECOPDAC 21—1; OEPA—1
Renal tumor	–	–	3	VCR + ACTD—1; doxorubicin + CTX—1; bevacizumab -1
Acute myeloid leukemia	–	–	2	Induction AIE—1; Ida-FLA—1
Non-Hodgkin lymphoma	-	–	2	R-CYM—1; VBL—1
Osteosarcoma	–	-	2	HD MTX—1; AP (week 1)—1
Retinoblastoma	1	Topotecan—1	–	–
Other (EWS, GCT, LCH, NET, HCC, SPN)	1	Sorafenib—1	4	VAC—1; VBL—1; gemcitabine 1 g/m2—1

Details on chemotherapy protocols are presented in Additional file 1

ACTD, actinomycin-D; CCNU, lomustine; CTX, cyclophosphamide; EWS, Ewing’s sarcoma; GCT, germ cell tumor; HCC, hepatocellular carcinoma; IFO, ifosfamide; LCH, Langerhans histiocytosis; MTX, methotrexate; NET, neuroendocrine tumor; SPN, solid pseudopapillary neoplasm; VCR, vincristine; VP-16, etoposide

In 14 cases (4 ALL, 2 CNS tumors, 1 Ewing’s sarcoma, 2 Hodgkin lymphoma, 1 hepatocellular cancer, 1 neuroblastoma, 1 retinoblastoma, 2 soft tissue sarcoma), the chemotherapy cycle was interrupted or prematurely stopped after SARS-CoV-2 infection was recognized; however, only 4 patients were symptomatic, and no COVID-19 attributable complications were observed in this group. In summary, chemotherapy in the period of COVID-19 diagnosis was associated with life-threatening complications in 1 case of COVID-19 pneumonia and severe GI bleeding, 2 cases of respiratory tract infection (COVID-19 not confirmed), 2 cases of mechanical ventilation in the ICU, and 2 cases of oxygen therapy. A fatal outcome of COVID-19 occurred in 2 patients, one of whom was heavily pretreated independent of SARS-CoV-2 complications.

After recovery from COVID-19, systemic therapy was continued, but delay in the next chemotherapy cycle was observed in 91 of 156 cases, with a median of 14 days (range 2–105 days) (Fig. 3D). Among survivors, a delay above 28 days was observed in 6 patients (6.5%). The primary diagnosis was not associated with statistically significant differences in chemotherapy delay (Fig. 3E).

## Discussion

COVID-19 has profoundly changed the approach to oncology. The first published reports raised warnings about the high risk of COVID-19-associated mortality in pediatric cancer patients [11]. Moreover, early Italian and US data suggested a reduced likelihood of pediatric cancer patients accessing referral centers, both because of a decrease in in-person primary care visits and the reluctance of families to risk exposing children to the virus, resulting in worse chances of a timely diagnosis [12, 13]. According to the study by Graetz et al., changes to cancer care delivery include reduced surgical care, blood product shortages, chemotherapy modifications, and interruptions to radiotherapy [14].

The introduction of new sanitary principles of screening, isolation guidelines and the use of personal protection equipment has helped to re-establish pediatric oncology services in the time of COVID-19 and adapt to new circumstances [15–17]. In Poland, healthcare personnel, who tested positive for SARS-CoV-2, was not allowed to attend work and was strictly isolated (for symptomatic cases: 10 days after symptom onset, plus at least 1 additional day without symptoms including without fever and without respiratory symptoms; for asymptomatic cases: 10 days after positive test for SARS-CoV-2). The caregivers/parents who tested positive were also isolated from their negative children, however due to social and logistic reasons the isolation could not be achieved in all cases. There were a few known cases during autumn 2020 COVID-19 "wave" in which negative children treated for cancer were hospitalized in one isolation room with their positive parent. These situations were exceptional—caused by the imperative need of treatment that could not be delayed (for example new diagnosis of leukemia, live saving chemotherapy for tumor progression or neutropenic fever) and no other possible options of providing childcare during hospitalization. In these rare situations, the caregivers were instructed how to minimalize the risk of infection transmission to the child (masks, disinfection).

After initial problems, positive changes emerged, such as value in cancer care, digital communication, convenience, inclusivity and cooperation, decentralization of cancer care, acceleration of policy change, human interactions, hygiene practices, health awareness and promotion and systems improvement [18]. A very large number of papers related to COVID-19 (at the moment of manuscript preparation over 180 thousand positions indexed in PubMed) is associated with variable quality of data sourcing and analysis and low practical usefulness in clinical settings.

Early SARS-CoV-2 surveillance in 2020 did not reveal a notable incidence in Poland, which might have been a consequence of both different epidemiology and diagnostic limitations [19]. Indeed, Polish data showed only 8 SARS-CoV-2-positive pediatric cancer patients by July 1, 2020 [20]. It must be noted that due to regulatory reasons, the availability of SARS-CoV-2 testing in Poland until June 2020 was insufficient, and many patients with symptoms of respiratory symptoms were not tested, even though testing for the other viral pathogens was carried out. This may be associated with underdiagnosis of COVID-19 in the pediatric population in the first half of 2020 because preliminary seroprevalence studies in June and September 2020 indicated the nationwide presence of anti-SARS-CoV-2-S antigen antibodies in up to 9% of the pediatric population (data unpublished). In contrast, the system was better equipped for viral diagnostics in autumn 2020, and the high incidence and morbidity of the late year peak were appropriately recorded. A typical clinical problem in children undergoing chemotherapy is diagnostics for neutropenic fever after chemotherapy, and patients with this condition are likely to be admitted to the hospital, especially if a central venous catheter is present. In patients diagnosed with SARS-CoV-2 infection, there is no correlation between fever and ANC value. These observations suggest that the role of the immune response in clinical presentation during the SARS-CoV-2 replication phase is still unclear. In 2020, SARS-CoV-2 PCR negativity during bone marrow aplasia after allogeneic hematopoietic stem cell transplantation was reported by the Polish transplant team, confirming the possibility of paucisymptomatic infection in a severely immunocompromised patient [21].

The role of the immune system in SARS-CoV-2 infection is not clear, and conflicting evidence exists regarding the connection among virus replication, the inflammatory response and tissue damage. The hypothesis of morbidity and mortality in COVID-19 as a consequence of excessive tissue damage in the mechanism of cytokine release syndrome is supported by high plasma levels of inflammatory cytokines in severely ill COVID-19 patients [22]. The decreased lymphocyte count and expression of exhaustion markers can be a consequence of CRS and correlate with a severe clinical course [23, 24]. Overall, the hypothesis of immune-mediated tissue damage leading to irreversible and fatal lung injury has had meaningful therapeutic consequences. Suppression of an excessive inflammatory response has been evaluated in COVID-19 patients treated with corticosteroids and anti-cytokine drugs. In a study by Wu et al., treatment with methylprednisolone in patients with acute respiratory distress syndrome (ARDS) decreased the risk of death (HR 0.38; 95% CI 0.20–0.72) [25]. The significance of interleukin-6 (IL6) is particularly interesting because of its key role in CRS and because elevated IL6 concentrations are associated with poor prognosis [26].

The SARS-CoV-2 PCR test does not distinguish between the presence of infectious virus particles and noninfectious nucleic acids [27]. Indeed, a positive PCR result reflects only the detection of SARS-CoV-2 RNA and does not necessarily indicate the presence of viable virus [28]. This is why the current strategy of isolation ending is fulfilment of the criteria of the time from SARS-CoV-2 infection identification and resolution of the clinical symptom period but does not require two consecutive negative PCR test results (as required at the beginning of the COVID-19 pandemic). PCR test results may still be used when there is a need for earlier isolation ending, but after the recommended isolation period is fulfilled, their usefulness is negligible.

There are data suggesting that although patients with SARS-CoV-2 infection might have detectable SARS-CoV-2 RNA for up to 83 days, no functional virus can be isolated beyond the ninth day after symptom onset, despite persistently high viral RNA loads [29]. The risk of having a positive SARS-CoV-2 culture is estimated to be three times higher in immunocompromised patients than in others, which suggests that immunocompromised patients may shed SARS-CoV-2 for prolonged periods [30]. Nevertheless, as culturing of SARS-CoV-2 was not performed in our study, the clinical implications of assessment of the length of positive PCR test results cannot be established. The correlation between interruptions in treatment resulting in decreased chemotherapy dose density and prognosis in pediatric cancer is unquestionable, but clear evidence is rarely published [31, 32]. Regardless, in many cases, the decision to continue chemotherapy after its disruption caused by SARS-CoV-2 infection was delayed by waiting for negative PCR results. Thus, the priority of pediatric cancer treatment was modified by the imperative of SARS-CoV-2 infection containment. However, the position of the pediatric hematology and oncology community in SARS-CoV-2 infection changed over time, and a course for minimizing delays and timely therapy was adopted. According to our data, chemotherapy can be administered shortly after SARS-CoV-2 infection diagnosis, provided that a patient does not display severe manifestations of COVID-19, which must be treated appropriately and can be associated with poor outcomes.

## Conclusions

Over the last few months, the community of pediatric oncologists has adapted to extraordinary circumstances and established mechanisms of coping with the COVID-19 pandemic. The situation can be expected to improve as a consequence of post-infection immunity and vaccinations against SARS-CoV-2, which should further protect patients, their families and medical caretakers [33]. Anti-SARS-CoV2 IgG status is not routinely tested after infection, but studies in common population show high diversity in seroprevalence in COVID-19 convalescents. An ongoing study in Polish pediatric cancer patients shows silent seroconversion ensuing the SARS-CoV-2 incidence in general population (data unpublished).

At the end of December 2020, the mRNA COVID-19 vaccines were introduced into public use in Poland, and since May 2021 were recommended in patients undergoing chemotherapy and after HCT [33]. Due to extreme vulnerability of cancer patients to COVID-19, children above the age of 12 years can be vaccinated 3 to 7 days after chemotherapy, but specific regulations apply to HCT recipients depending on graft-versus-host symptoms and antecedent lymphodepleting therapies. The effect of vaccinations is expected to reduce the risk of therapy interruptions and incidence of COVID-19 complications.

The long-term prognosis of treated patients is unclear due to the short observation period. The effects on pediatric cancer curability must be analyzed in the future, but according to our observations, interruptions in therapy are common, and current data indicate that the issue of suboptimal therapy in COVID-19 survivors can be addressed in the future.

## Supplementary Information

**Additional file 1**. List of chemotherapy protocols.

## Data Availability

The datasets used and/or analyzed during the current study are available from the corresponding author on reasonable request.

## Abbreviations

ACTD: Actinomycin-D
ALL: Acute lymphoblastic leukemia
ANC: Absolute neutrophil count
CCNU: Lomustine
CI: Cumulative incidence
COVID-19: Coronavirus disease 2019
CTX: Cyclophosphamide
ECDC: European Centre for Disease Prevention and Control
EWS: Ewing’s sarcoma
GCT: Germ cell tumor
HCC: Hepatocellular carcinoma
HCT: Hematopoietic cell transplantation
IFO: Ifosfamide
LCH: Langerhans histiocytosis
MTX: Methotrexate
NAT: Nucleic acid testing
NET: Neuroendocrine tumor
OS: Overall survival
PCR: Polymerase chain reaction
PICU: Pediatric intensive care unit
SARS-CoV-2: Severe acute respiratory syndrome coronavirus 2
SPN: Solid pseudopapillary neoplasm
UPN: Unique patient number
VCR: Vincristine
VP-16: Etoposide
WBC: Whole blood count
