# Supplementary material for: COVID-19 in pediatric cancer patients is associated with treatment interruptions but not with short-term mortality: a Polish national study
Source: J Hematol Oncol. 2021 Oct 11;14:163. doi: 10.1186/s13045-021-01181-4 (PMC8503711; doi:10.1186/s13045-021-01181-4)
Supplement: Supplementary file 1 — Additional file 1. List of chemotherapy protocols. [file 13045_2021_1181_MOESM1_ESM.docx]

**Supplemental file 1**

**Chemotherapy cycles**

**Soft tissue sarcomas:**

1. VACA - ACCTTIVE: Vincristine (VCR): 1,5 mg/m2 – day 1

Cyclophosphamide (CTX): 1200 mg/m2 – day 1

Doxorubicin: 20 mg/m2 – days 1, 2, 3

1. Gemcitabin + Vinorelbin: Gemcitabine: 900 mg/m2 – day 1 and 8

Vinorelbine: 25 mg/m2 – day 1 and 8.

1. I2VAd: VCR: 1,5 mg/m2 – day 1

Ifosfamide (IFO): 3 g/m2 – day 1 and 2

Doxorubicin: 2 x 20 mg/m2/day – day 1 and 2

1. I3VA: VCR: 1,5 mg/m2 – day 1

Actinomycin-D (ACTD): 1,5 mg/m2 – day 1

IFO: 3 g/m2 – day 1, 2, 3

1. Idarubicin+trofosfamide: Idarubicin: 5mg/m2 – day 1, 4, 7, 10

Trofosfamide: 150 mg/m2 – days 1-10

1. I2VE VCR: 1,5 mg/m2 – day 1

IFO: 3 g/m2 – day 1 and 2

Etoposide (VP-16): 150 mg/m2 – day 1 and 2

1. I2VA VCR: 1,5 mg/m2 – day 1

ACTD: 1,5 mg/m2 – day 1

IFO: 3 g/m2 – day 1 and 2

1. TECC Topotecan: 1 mg/m2 – days 1, 2, 3, 4

Carboplatin: 150 mg/m2 – days 1, 2, 3, 4

1. VAC VCR: 1,5 mg/m2 – day 1

ACT-D: 1,5 mg/m2 – day 1

CTX: 20 mg/kg – day 1

1. SPN Gemcitabine: 1 g/m2 – day 1, 8, 15

**CNS Tumors**

1. VCR+VP16+CTX: VCR: 1,5 mg/m2 – day 1

CTX: 1500 mg/m2 – day 1

VP-16: 100 mg/m2 – day 1, 2, 3

1. VCR+Cisplatin: VCR: 1,5 mg/m2 – day 1

Cisplatin: 75 mg/m2 – day 1

1. VCR: VCR: 1,5 mg/m2 – day 1
2. VCR+VP16+CTX: VCR: 1,5 mg/m2 – day 1

CTX: 1500 mg/m2 – day 1

VP-16: 100 mg/m2 – days 1, 2, 3

1. VCR+Cisplatin+CCNU: VCR: 1,5 mg/m2 – day 1

Cisplatin: 75 mg/m2 – day 1

CCNU: 75 mg/m2 – day 1

1. Protocol 4 for LGG: week 8: VCR: 1,5 mg/m2 – day 1

week 29: VCR: 1,5 mg/m2 – day 1

Carboplatin: 550 mg/m2 – day 1

1. VBL: Vinblastine 6 mg/m2 – day 1
2. Protocol 1 HRG
   1. VCR: 1,5 mg/m2 – day 1

VP-16: 100 mg/m2 – days 1, 2, 3

Carboplatin: 500 mg/m2 – day 1, 2

- 1. VP-16: 60 mg/m2 – days 1, 2, 3, 4, 5

IFO: 900 mg/m2 – days 1,2 ,3 4, 5

1. Temozolomide + Cisplatin: Temozolomide: 150 mg/m2 – days 1, 2, 3, 4, 5

Cisplatin: 20 mg/m2 – day 1, 2, 3, 4, 5

**Hodgkin Lymphoma**

1. OEPA: Prednisone: 60 mg/m2/day – days 1-15

VCR: 1,5 mg/m2 - days 1, 8, 15

Doxorubicin: 40 mg/m2 – days 1 and 15

VP-16: 125 mg/m2 – days 1, 2, 3, 4, 5

1. COPDAC: Prednisone: 40 mg/m2/day – days 1 – 15

Dacarbazine: 250 mg/m2 – days 1, 2, 3

VCR: 1,5 mg/m2 - days 1, 8

CTX: 500 mg/m2 – days 1, 8

1. DECOPDAC-21: Prednisone: 40 mg/m2/day – days 1 – 8

Dacarbazine: 250 mg/m2 – days 1, 2, 3

VCR: 1,5 mg/m2 - days 1, 8

CTX: 625 mg/m2 – days 1, 8

VP-16: 100 mg/m2 – days 1, 2, 3

Doxorubicin: 25 mg/m2 – day 1

1. AVD: Doxorubicin: 25 mg/m2 – days 1, 15

VBL: 6 mg/m2 – days 1, 15

Dacarbazine: 375 mg/m2 – days 1, 15

**Osteosarcoma:**

1. HD-MTX: Methotrexate (MTX) 12 g/m2 – day 1
2. AP: Doxorubicin: 75 mg/m2 – day 1, 2

Cisplatin: 120 mg/m2 – day 1, 2, 3

**Other:**

1. Ewing’s sarcoma VAC VCR: 1,5 mg/m2 – day 1

ACT-D: 0,75 mg/m2 – day 1, 2

CTX: 1500 mg/m2 – day 1.

1. Ida-FLA Fludarabine: 30 mg/m2 – days 1-4

Cytarabine: 2000 mg/m2 – days 1-4

Idarubicin: 12 mg/m2 – days 2-4

1. COJEC-A Carboplatin: 750mg/m² - day 1

VP-16: 175mg/m² - day 1,2

VCR: 1.5mg/m² - day 1

1. R-CYM Cytarabine: 100 mg/m2/day, days 2-6

MTX: 3000 mg/m2, day 1

Rituximab: 375 mg/m2, day 1
